# Ionic mechanisms maintaining action potential conduction velocity at high firing frequencies in an unmyelinated axon

**DOI:** 10.14814/phy2.12814

**Published:** 2016-05-24

**Authors:** Kevin P. Cross, R. Meldrum Robertson

**Affiliations:** ^1^ Centre for Neuroscience StudiesQueen's UniversityKingstonOntarioCanada; ^2^ Department of BiologyQueen's UniversityKingstonOntarioCanada

**Keywords:** ADP, computer model, insect, locust DCMD, persistent sodium

## Abstract

The descending contralateral movement detector (DCMD) is a high‐performance interneuron in locusts with an axon capable of transmitting action potentials (AP) at more than 500 Hz. We investigated biophysical mechanisms for fidelity of high‐frequency transmission in this axon. We measured conduction velocities (CVs) at room temperature during exposure to 10 mmol/L cadmium, a calcium current antagonist, and found significant reduction in CV with reduction at frequencies >200 Hz of ~10%. Higher temperatures induced greater CV reductions during exposure to cadmium across all frequencies of ~20–30%. Intracellular recordings during 15 min of exposure to cadmium or nickel, also a calcium current antagonist, revealed an increase in the magnitude of the afterhyperpolarization potential (AHP) and the time to recover to baseline after the AHP (Medians for Control: −19.8%; Nickel: 167.2%; Cadmium: 387.2%), that could be due to a T‐type calcium current. However, the removal of extracellular calcium did not mimic divalent cation exposure suggesting calcium currents are not the cause of the AHP increase. Computational modeling showed that the effects of the divalent cations could be modeled with a persistent sodium current which could be blocked by high concentrations of divalent cations. Persistent sodium current shortened the AHP duration in our models and increased CV for high‐frequency APs. We suggest that faithful, high‐frequency axonal conduction in the DCMD is enabled by a mechanism that shortens the AHP duration like a persistent or resurgent sodium current.

## Introduction

In axons, supernormal and subnormal conduction occur when action potential (AP) propagation velocity is increased or decreased relative to an initial AP in previously inactive membrane (Bucher and Goaillard 2011). Supernormal and subnormal conduction can alter spike timing, which can affect temporal coding strategies in which information is stored in instantaneous frequencies and delays (Dayan and Abbot 2001). Supernormal conduction commonly occurs for low‐frequency APs between 50 and 150 Hz and is caused by discharge of the membrane capacitance that can be altered by ion channels (Barrett and Barrett 1982; Bostock et al. 2003; Bucher and Goaillard 2011). Subnormal conduction commonly occurs during bursts of high‐frequency APs as the relative refractory period of the preceding AP decreases the number of active sodium channels available for the subsequent AP and can compromise transmission by high‐frequency firing neurons.

High‐frequency firing neurons are involved in many circuits in the mammalian nervous system. Cortical fast‐spiking interneurons, thalamic relay neurons and globular bushy cells are all capable of following frequencies >100 Hz (Steriade et al. 1993; Rudy et al. 1999; Rhode 2008). Mechanisms for producing high‐frequency APs have been well studied and include K_v_3 potassium currents, which provide fast repolarization of APs (Rudy and McBain 2001), and T‐type calcium currents, which provide a prolonged depolarization for high‐frequency bursting (Cain and Snutch 2013). However, transmission of high‐frequency APs by axons is poorly understood due to their small diameters making them inaccessible to direct study. Instead, invertebrate giant axons and computational modeling have been primarily used to explore axon dynamics (Debanne 2004). Furthermore, little is known about axonal mechanisms that counteract or enhance supernormal or subnormal conduction.

The descending contralateral movement detector (DCMD) neuron in the locust provides a model axon for studies of high‐frequency transmission. It is a giant axon that relays visual information at high velocities from the lobula giant movement detector (LGMD) neuron in the brain to neurons in the thoracic ganglia (O'Shea et al. 1974). Its response to a looming visual stimulus has been well characterized and is involved with predator evasion (Gabbiani et al. 1999, 2001, 2002; Gray et al. 2001; Fotowat and Gabbiani 2007; Fotowat et al. 2011). It reliably transmits high‐frequency APs to motoneurons to trigger escape behavior (Santer et al. 2006; Fotowat et al. 2011) and at normal operating temperatures is capable of frequencies >500 Hz (Money et al. 2005).

**Figure 1:**
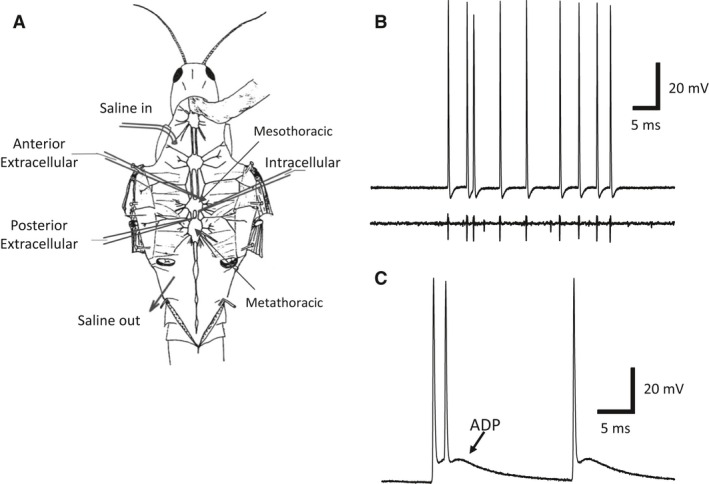
Experimental set‐up. (A) The semiintact preparation of showing the meso‐ and metathoracic ganglia and the placement of the extracellular and intracellular electrodes. For high‐temperature experiment, heated saline was pumped in near the animal's head and flowed out near the abdomen. (B) An intracellular recording from the DCMD axon (top) and its equivalent extracellular trace (bottom) recorded simultaneously. (C) A recording from the DCMD illustrating the presence of the afterdepolarizing potential (ADP) typical of heat‐shocked animals at high temperature with a resting membrane potential of ~−70 mV.

In the DCMD, elevated temperatures increase maximal firing frequency and produce an afterdepolarizing potential (ADP) that is modulated by temperature and a preconditioning heat shock (Money et al. 2005) (Fig. 1C). By temporarily depolarizing the membrane potential above its resting value, the ADP is believed to improve excitability in the DCMD axon (Money et al. 2005) and similar ADPs are present in rat and frog axons where they contribute to supernormal conduction (Bowe et al. 1987). Several currents can produce similar ADPs including T‐type calcium currents, persistent and resurgent sodium currents, and calcium‐activated nonselective calcium current (Bean 2007).

We investigated the mechanisms that allow for high‐frequency conduction in the DCMD axon. We began by hypothesizing that a T‐type calcium current underlies the ADP generated in the axon to enable high‐frequency conduction. To test this, we exposed the DCMD axon to divalent cations, cadmium and nickel, antagonists of T‐type calcium currents. Divalent cations significantly reduced conduction velocity (CV), particularly at high frequencies in the DCMD axon, and increased the afterhyperpolarization (AHP) magnitude. Removal of extracellular calcium did not mimic the effects of the divalent cations on CV, suggesting the effects of the divalent cation channel were not mediated by a T‐type calcium current. Computational models confirm that currents that shorten the AHP magnitude and duration, like the persistent and resurgent sodium currents, can improve high‐frequency conduction by the axon.

## Materials and Methods

### Animal preparation

Experiments were conducted on adult male locusts (*Locusta migratoria*) aged 2–5 weeks past their final molt. They were raised in a crowded colony maintained at Queen's University with a 12 h:12 h photoperiod. Animals were fed wheat grass and a mixture of bran, skim milk powder and yeast daily. Cage temperatures were elevated above room temperature (25°C) to 30°C using 60‐W incandescent bulbs during the light cycle.

Locusts were dissected as previously described (Robertson and Pearson 1982). Briefly, the animals were pinned open to expose the meso‐ and metathoracic ganglia and a metal plate was placed under the thoracic ganglia to improve stability. Standard locust saline bathed the thoracic ganglia and was composed of (in mmol/L): 147 NaCl, 10 KCl, 4 CaCl2, 3 NaOH, and 10 HEPES. Nerve roots were cut close to the mesothoracic ganglion to allow for drug delivery and a chlorided silver wire was placed in the abdomen and grounded (Fig. 1A).

### Electrophysiological recording

For temperature‐controlled experiments, we continuously flowed heated saline into the thoracic cavity using a peristaltic pump (Peri‐star) and a proportional temperature controller (Scientific Systems Design). A BAT‐12 thermometer (PhysiTemp) was connected to a type T thermocouple wire of diameter ~ 0.3 mm and was used to monitor the saline temperature by placing the thermocouple near the mesothoracic ganglion.

Glass suction electrodes were placed on the dorso‐medial surface of the connective between the pro‐ and mesothoracic ganglia and between the meso‐ and metathoracic ganglia. Signals were amplified and filtered using an AM‐Systems AC Differential Amplifier model 1700 and digitized with an Axon Instruments Digidata 1440A digitizer. Recordings were made with AxoScope 10.3 (Molecular Devices) and were analyzed using the Clampfit module of pClamp 10.2 (Molecular Devices) software. The DCMD activity was easily distinguished from background spiking in the connective by its large spike amplitude and its robust response to visual stimuli (extracellular recording in Fig. 1B).

Intracellular electrodes were pulled from borosilicate glass and filled with 3 mol/L KCl, giving them a tip resistance between 20 and 40 MΩ. Recordings were amplified and filtered with an AM‐Systems Neuroprobe Amplifier model 1600 and digitized at 83 kHz by a Digidata 1440A. The amplifier's DC offset was zeroed relative to the bath before penetration of DCMD and the penetration was made just caudal to the mesothoracic ganglion on the dorso‐medial surface (Fig. 1A). The temperature was maintained at 35°C and the resting membrane potential was fixed to −65 mV, near the natural resting membrane potential for the DCMD at 35°C (Money et al. 2005) allowing for comparisons of AP parameters.

### Pharmacology

All chemicals used were obtained from Sigma‐Aldrich Canada. For cadmium and nickel experiments, 10 mmol/L of CdCl_2_ or NiCl_2_ in standard locust saline was applied for 50 min or until conduction failed. We chose such a high concentration of the divalent cations to ensure timely delivery, as the cut nerve roots that facilitate drug delivery are small and reside on the periphery of the ganglion, whereas the DCMD traverses the medial surface. Longer bath applications are not ideal, particularly with intracellular recordings which last only ~15–20 min when the preparation is exposed to divalent cations.

We used two types of calcium‐free saline; both were standard locust saline without calcium, however, one contained an additional 4 mmol/L of MgCl_2_ to maintain the divalent cation concentration. High calcium saline had an additional 4 mmol/L of CaCl_2_ added to standard saline to double the calcium concentration. In all experiments, an initial recording of the DCMD activity was made before exposure and every 5 min thereafter.

In TTX (tetrodotoxin) experiments, TTX was dissolved in 1% acetic acid buffer with a pH of 4.75 to produce a concentration of 300 nmol/L.

### Data analysis

Analysis of extracellular measurements included the instantaneous frequency of APs determined by the interspike interval, and the CV, which is proportional to the reciprocal of the time taken to travel from the anterior to the posterior electrode. CVs were calculated relative to the first AP in the recording to control for slight variation in the distance between the electrodes. Metrics used to analyze the intracellular recordings were extracted by custom Python scripts using the StimFit library (Guzman et al. 2014).

When comparing multiple groups we used a one‐way ANOVA with Holm–Sidak pairwise multiple comparisons or one‐way ANOVA on Ranks with Student–Neuman–Keuls method pairwise multiple comparisons. When comparing multiple groups over time we used a two‐way ANOVA or a two‐way repeated measures (RM) ANOVA with Holm–Sidak pairwise multiple comparisons. Statistical tests were completed with SigmaPlot 11.0 (Systat Software) and significance was defined as *P* < 0.05. Data are reported as either mean ± standard error (SE) for normally distributed data or median (Mdn) and interquartile range (IQR) for nonparametric data.

Statistical significance (*P* < 0.05) in plots is denoted by a letter. Columns with different letters indicate statistically different groups, whereas columns with the same letter are not significantly different.

### Single‐compartment model

We used a basic model whose current kinetics were described by Wang (1998) and which has been used previously to describe the LGMD axon (Peron and Gabbiani 2009). We modified the model by including only the transient sodium, delayed‐rectifying potassium and leakage currents. We changed the reversal potential for the sodium current (*V*_Na_) to 45 mV to better fit the experimental data and changed the leakage conductance 0.3 mS/cm^2^ as explained in the results section. We also temporally evolved the sodium activation gate $dm/dt=\left(m-m_{\infty }\right)/\tau_{m}$, where m is the activation gate, $m_{\infty }=\alpha_{m}/\left(\alpha_{m}+\beta_{m}\right)$ and *τ*_m_ = 1/(*α*_m_ + *β*_m_) as opposed to Wang (1998) which assumed $m=m_{\infty }$. Functions for *α*_m_ and *β*_m_ are identical to those in Wang (1998).

We included a persistent sodium current $I_{\mathrm{Nap}}=G_{\mathrm{Nap}}m_{p}\left(V_{m}-V_{\mathrm{Na}}\right)$ where *G*_Nap_ is the persistent sodium conductance set to either 0.2 or 0.3 mS/cm^2^, *V*_m_ is the membrane potential, and m_p_ is the activation gate that evolved similar to the transient sodium current mentioned earlier. It was governed by the activation gate's steady‐state value $m_{p\infty }=1/\left\{1+\text{exp}\left[-0.17\left(V_{m}+17\right)\right]\right\}$ (Smith et al. 1998) and time constant *t*_p_ = 1 msec (Chatelier et al. 2010).

We also included simulations where we added a resurgent sodium current to our base model (no persistent sodium current) *I*_Nar_ = *G*_Nar_ *m*_r_ *h*_r_(*V*_m_ − *V*_Na_) where *G*_Nar_ is the resurgent sodium conductance set to 1.0 or 1.5 mS/cm^2^, *m*_r_ and *h*_r_ are the activation and inactivation gate, respectively. Kinetics for the activation and inactivation gate are from D'Angelo et al. (2001).

Temporal evolution of the membrane potential obeyed the first‐order kinetic

(1) $$C_{m}\frac{dV_{m}}{dt}=-I_{\mathrm{Na}}-I_{k}-I_{L}-I_{\mathrm{Nap}}-I_{\mathrm{Nar}}+I_{\mathrm{stim}}$$

where *C*_m_ is the membrane capacitance set to 1 *μ*F/cm^2^, *I*_Na_ is the transient sodium current, *I*_K_ is the potassium current, *I*_L_ is the leakage current, *I*_stim_ is the stimulus current. Equation (1) and the current gating variables were both solved by Euler's method with a time step of 10^−3^ msec.

To test frequency‐dependent fidelity, the single compartment was stimulated with a 150 *μ*A/cm^2^ current for 0.2 sec for 100 spikes.

### Multicompartment model

The DCMD was modeled as a 1‐cm‐long cylinder with a fixed radius of 10 *μ*m without branching. We partitioned the axon into 100 compartments each of length 100 *μ*m and having passive and active properties identical to the single current model. Temporal evolution of the membrane potential of compartment *μ* now requires two additional terms to equation (1)

(2) $$C_{m}\frac{dV_{m}}{dt}=-I_{\mathrm{Na}}-I_{k}-I_{L}-I_{\mathrm{Nap}}-I_{\mathrm{Nar}}+I_{\mathrm{stim}}+G_{\mu ,\mu +1}\left(V_{\mu +1}-V_{\mu }\right)+G_{\mu ,\mu -1}\left(V_{\mu -1}-V_{\mu }\right)$$

where *G*_*μ*,*μ*+1_ is the conductance from compartment *μ*+1 to *μ* and, similarly, *G*_*μ*,*μ*‐1_ is the conductance from *μ*−1 to *μ*. We fixed all intercompartmental conductances to 50 mS/cm^2^. As described in Dayan and Abbot (2001), we used the Crank–Nicholson method to solve equation (2) and Euler's method to solve the channel gating variables. We used a time step of 5 × 10^−5^ msec.

We stimulated the first compartment in the model with a current injection of 500 *μ*A/cm^2^ for 0.2 msec to elicit APs. The timing pattern for the stimulus was produced from the DCMD activity of 8 locusts in response to a looming visual stimulus.

## Results

### Divalent cations reduced CV in the DCMD

**Figure 2:**
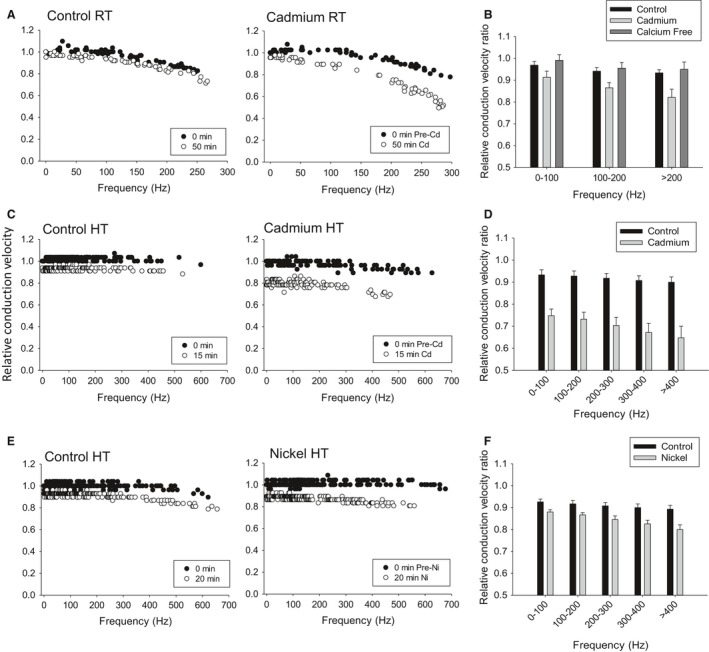
Divalent cations reduce conduction in the DCMD axon. (A) Relative CV profiles show decreased CV after 50 min of exposure to 10 mmol/L of cadmium at room temperature (RT). (B) Relative conduction velocity ratios show cadmium had a significant main effect, reducing velocity while removing extracellular calcium (Calcium Free) caused no changes (Controls: *n* = 9; Cadmium: *n* = 8; Calcium free: *n* = 5; Two‐way ANOVA with Holm–Sidak pairwise multiple comparisons, df = 2, *F* = 13.2, *P* < 0.001). (C) Relative CV profiles after 15 min exposure to cadmium at 35°C (HT). (D) Relative CV ratios show cadmium at 35°C had a significant main effect after 15 min (Control: *n* = 6; Cadmium: *n* = 7; Two‐Way ANOVA, df = 1, *F* = 108.0, *P* < 0.001). (E) Relative CV profiles after 20 min exposure to nickel at 35°C. (F) Relative CV ratios show nickel at 35°C had a significant main effect after 20 min (Control: *n* = 7; Nickel: *n* = 6; Two‐Way ANOVA, df = 1, *F* = 41.7, *P* < 0.001).

We investigated the hypothesis that the DCMD's axon expresses a calcium current that improves high‐frequency conduction. We used 10 mmol/L of cadmium, a nonselective calcium current blocker, and calcium‐free saline with 1 mmol/L EGTA, a calcium chelator. They were bath applied for 50 min at room temperature (Fig. 2A). Cadmium produced a significant reduction in CVs with a larger effect size for high‐frequencies (~10%), whereas calcium‐free saline had no effect (Fig. 2B). Given this high‐frequency slow‐down, we elevated the temperature to 35°C, which is known to cause hyperexcitability in the DCMD's axon and increases the magnitude of the ADP (Money et al. 2005). We added 10 mmol/L of cadmium at 35°C, which significantly decreased CV (~20–30%) after only 15 min of exposure (Fig. 2C and D). We also used 10 mmol/L nickel, another nonselective calcium current blocker, and found reductions in CV similar to the effect of cadmium after 20 min of exposure, however with a lower effect size (~5–10%) (Fig. 2E and F). In six of seven cadmium experiments at high temperatures and six of six nickel experiments, activity was lost in the posterior electrode indicating transmission failure, whereas five of five controls that were allowed to run for 1 h maintained activity in both electrodes. On average, cadmium trials lost posterior activity after 24.2 ± 1.5 min of exposure and nickel after 32.5 ± 1.1 min.

### Divalent cation's effects on the DCMD's AP

**Figure 3:**
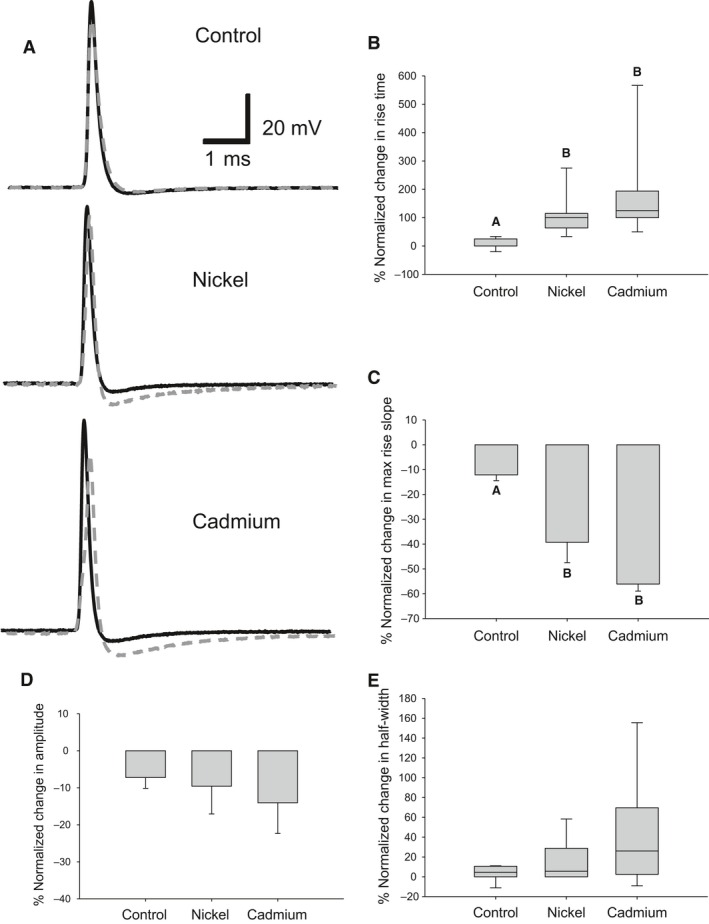
Divalent cations reduce rising component of the AP at high temperature. (A) Representative APs at 35°C before (black solid) and 15 min after exposure (gray dashed). Clear changes to the AP can be seen on the rising phase during cadmium and nickel exposure. (B) Cadmium and nickel significantly increased the rise time of the AP (Controls: *n* = 8; Cadmium: *n* = 8; Nickel: *n* = 8; One‐way ANOVA on Ranks with Student–Neuman–Keuls method pairwise multiple comparisons, df = 2, *H* = 16.14, *P* < 0.001) (C) and decreased the maximum rise slope as compared to controls (One‐way ANOVA with Holm–Sidak pairwise comparisons, df = 2, *F* = 15.3, *P* < 0.001). (D) Cadmium and nickel did not affect the AP amplitude (E) or the half‐width. (C, D) Data are plotted as mean and SE. (B, E) Data are plotted as median and upper and lower quartile. Letters indicate significant differences (*P* < 0.05).

We then used intracellular electrodes to investigate how the divalent cations modulated AP parameters in the DCMD's axon. We penetrated the DCMD near the mesothoracic ganglion and exposed it to 10 mmol/L cadmium or nickel for 15 min at 35°C. During exposure to cadmium or nickel, clear changes occurred on the rising slope of the AP indicating a possible compromise of the transient sodium current (Fig. 3A). Divalent cations are known to modulate the transient sodium channel by screening the negative surface charge near the transient sodium channel (Hille et al. 1975) or through direct interaction with the voltage sensor (Gilly and Armstrong 1982b). We characterized changes to the rising phase of the AP by the rise time, which is the duration of the rising phase between 10% and 90% of the peak of the AP, and the max rise slope, which is the maximum time derivative of the rising phase. Cadmium and nickel significantly increased the percentage normalized change (PNC) in rise time (Fig. 3B) (Control: *n* = 8, Mdn = 0.0%, IQR = 0.0, 25.0%; Nickel: *n* = 8, Mdn = 100.0%, IQR = 63.8, 115.0%; Cadmium: *n* = 8, Mdn = 125.0%, IQR = 100.0, 193.8%) and significantly decreased the PNC in max rise slope (Fig. 3C) (Control: −9.6 ± 4.9%; Nickel: −42.7 ± 4.7%; Cadmium: −51.8 ± 7.1%). However, changes to the rising slope did not correspond to a significant change in the PNC in AP amplitude (Fig. 3D) (Control: −7.6 ± 2.5%; Nickel: −13.2 ± 3.8%; Cadmium: −17.0 ± 5.3%) or the PNC in AP half‐width, which was defined as the full width at half maximum amplitude of the AP (Fig. 3E) (Control: Mdn = 4.5%, IQR = 0.0, 10.6%; Nickel: Mdn = 5.6%, IQR = 0.0, 28.8%; Cadmium: Mdn = 26.1%, IQR = 2.5, 69.7%), though there were trends for amplitude to be reduced and half‐width increased after treatment with divalent cations.

**Figure 4:**
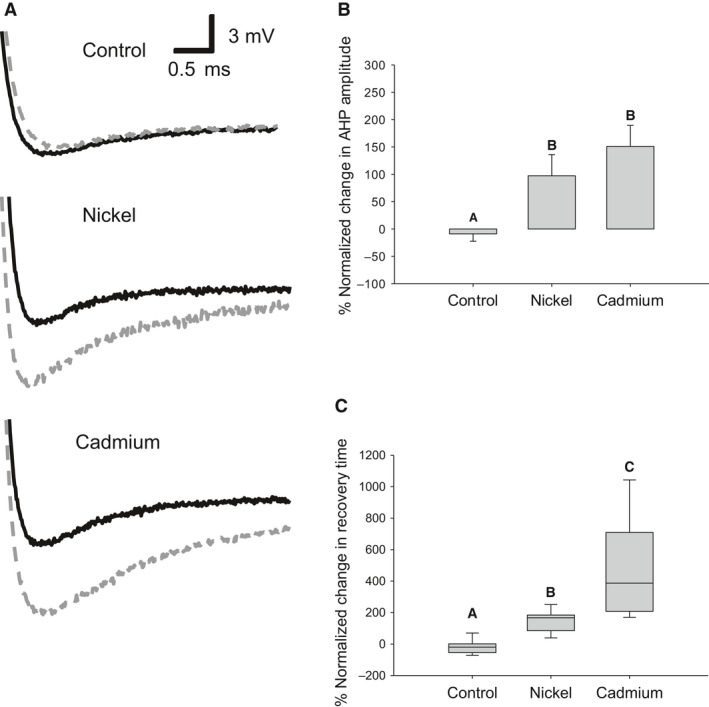
Divalent cations increase the AHP. (A) Close‐ups of the AP's AHPs before (black solid) and after the addition of cadmium or nickel for 15 min at 35°C (gray dashed). Controls continued to receive saline. (B) Cadmium and nickel both caused a significant increase in the size of the AHP amplitude (Controls: *n* = 8; Cadmium: *n* = 8; Nickel: *n* = 8; One‐way ANOVA with Holm–Sidak pairwise multiple comparisons, df = 2, *F* = 19.6, *P* < 0.001). (C) Cadmium and nickel significantly increased the recovery period from the AHP compared to nickel (One‐way ANOVA on Ranks with Student–Neuman–Keuls method pairwise multiple comparisons, df = 2, *H* = 18.7, *P* < 0.001). (B) Data are plotted as mean and SE. (C) Data are plotted as median and upper and lower quartile. Letters indicate significance (*P* < 0.05).

Recorded APs also showed an increase in the AHP magnitude and duration during divalent cation exposure (Fig. 4A). AHP changes were characterized by the AHP amplitude, which is the voltage difference of the AHP's minimum and the resting membrane potential (held at −65 mV), and the AHP recovery time, which is the time between the AHP's minimum and its recovery to the resting membrane potential. The PNC in AHP amplitude (Control: −13.3 ± 9.1%; Nickel: 113.4 ± 22.7%; Cadmium: 163.5 ± 26.0) and recovery time were significantly larger during divalent cation exposure (Fig. 4B and C) (Control: Mdn = −19.8%, IQR = −54.7, 1.5%; Nickel: Mdn = 167.2%, IQR = 84.9, 184.2%; Cadmium: Mdn = 387.2%, IQR = 207.8, 709.6%).

### Calcium saline manipulation's effects on CV in the DCMD

**Figure 5:**
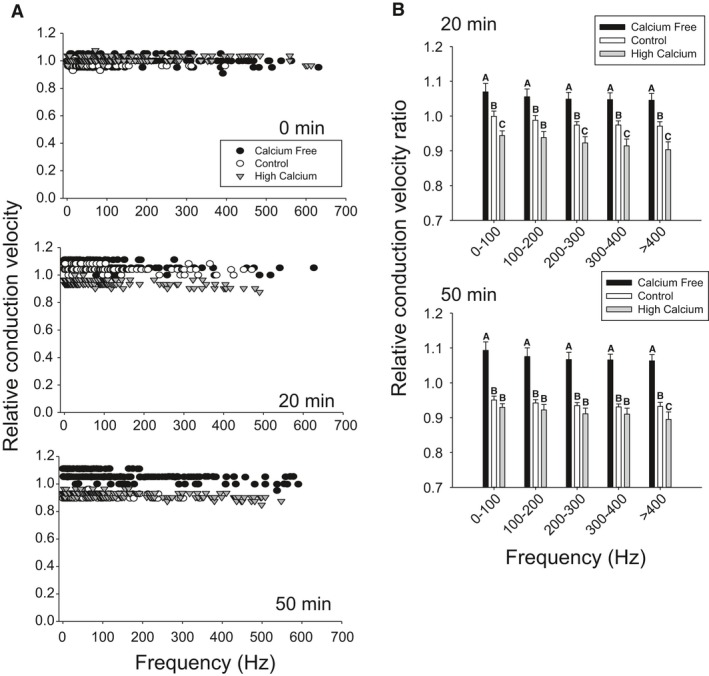
Calcium removal increases CV in the DCMD at high temperature. (A) Representative relative CV profiles at 35°C show opposite effects of calcium free (calcium‐free saline with 1 mmol/L EGTA) and high calcium (saline with 8 mmol/L calcium) at 20 and 50 min of exposure. (B) Calcium‐free saline (*n* = 6) produced a significant increase in CV across all frequencies compared to controls (*n* = 7) at 20 and 50 min (*P* < 0.05). High calcium (*n* = 5) produced an initial reduction in CV at 20 minutes across most frequencies but after 50 min, controls had slowed to equal high calcium in all but the >400 Hz band (Two RM ANOVA with Holm–Sidak pairwise multiple comparisons, df = 2, *F* > 18 for all frequencies, *P* < 0.001 for all frequencies). Data are plotted at mean and SE. Letters indicate significant differences (*P* < 0.05).

We further investigated the mechanism by which the divalent cations were modulating the DCMD's performance by manipulating the calcium concentration in the bath saline. We used a calcium‐free saline with 1 mmol/L EGTA to reduce the calcium gradient across the DCMD axon's membrane. We hypothesized that the reduced calcium gradient would decrease the calcium current, mimicking the block by divalent cation and decrease CV at high frequencies. We also used a high calcium saline (8 mmol/L calcium), which would increase the calcium gradient and amplify the calcium current and hypothesized that increased calcium current would increase CV at high frequencies. We did not expect the increase in divalent cation concentration in high calcium saline to modulate the transient sodium current greatly as it is a smaller concentration change (4 mmol/L) than our other experiments (10 mmol/L) and cadmium and nickel are >5 × more effective at transient sodium current modulation than calcium (Hanck and Sheets 1992). We exposed the DCMD to 50 min of calcium‐free or high calcium saline at 35°C. After 50 min, we found calcium‐free saline caused an increase in CV at all frequencies at the 20 and 50 min time points, whereas control and high calcium saline had decreased in CV (Fig. 5A and B). We also completed calcium‐free trials with magnesium substituted for calcium (4 mmol/L Mg) to correct for divalent screening of surface charges and found a similar increase in CV compared to controls (data not shown). These data suggest that the effects of the divalent cations on the DCMD's CV are not mediated by a calcium current.

### TTX's effects on the DCMD

Other currents are also capable of producing ADPs including the persistent and resurgent sodium current (Bean 2007; Bucher and Goaillard 2011), which can be modulated and blocked by divalent cations (Schafer et al. 1994; Li and Hatton 1996; Afshari et al. 2004; Yue et al. 2005) and may be responsible for AHP shortening. Furthermore, persistent sodium currents have been described in other invertebrate species including: squid giant axons (Clay 2002), honeybee Kenyon cells (Schafer et al. 1994), and cockroach aminergic neurons (Lapied et al. 1990). We attempted to determine if the current was due to a persistent or resurgent sodium channel using TTX, a sodium channel blocker. We exposed the DCMD to 300 nmol/L of TTX at 35°C and could continuously record extracellular activity in the nerve cord for the entire 50 min duration of the recording. Analysis of the relative CV ratio at 10 and 50 min after exposure (data not shown) by a Two‐Way RM ANOVA found a significant interaction effect across all frequency bins (Control: *n* = 4; TTX: *n* = 5; df = 1, *F* > 34, *P *≤ 0.002 for all frequency bins). Holm–Sidak pairwise multiple comparisons revealed no significant effect of TTX at 10 min (*P* > 0.45 for all frequency bins) and a significant reduction of ~15% in relative CV ratio compared to controls after 50 min (*P* < 0.008 for all frequency bins). Although these data mimicked the effects the divalent cations had on the DCMD conduction, they are confounded by possible effects the TTX may have had on the transient sodium channel. We attempted to follow up our CV experiments with intracellular measurements of the AP's AHP during TTX exposure. However, stability of the intracellular measurements were compromised after only 10 min of TTX exposure as reflected by a loss in measured AP amplitude. This was likely an artifact of the electrode losing its penetration as an AP amplitude decrease should result in CV reduction, which was absent after 10 min in our previous experiment. Uncoordinated and large muscle contractions in the abdomen that rapidly displace bath saline likely contributed to penetration loss of the intracellular recordings. Unfortunately, attempts with other sodium channel blockers would likely cause similar problems with recording quality and illustrates the difficulty of semiintact preparations. However, removal of the nerve cord containing the axon from the animal would be challenging, as the DCMD axon is identified in the connective by its characteristic response to visual stimulus requiring an intact visual system. Lastly, results from reduced extracellular sodium concentration would be difficult to interpret as the inevitable reduction in the driving force on the transient sodium current could impair the activation of potassium channels and lessen the magnitude of the AHP. Instead, we elect to explore mechanisms of AHP magnitude and their influence on CV through computer simulations.

### Computer simulations suggests AHP reduction improves high‐frequency

We built a single‐compartment model of the DCMD axon to investigate the effect AHP shortening may have on firing fidelity and CV in an axon. The model contained a transient sodium current, delayed‐rectifying potassium current, and a leakage current. We also included a persistent or resurgent sodium current to shorten the AHP as we feel they are the best candidates underlying the effects of divalent cations on the AHP (see Discussion). We used a large leakage conductance of 0.3 mS/cm^2^ as it decreased the AHP recovery time to better match the physiological data and is similar to reported leakage conductance for squid giant axon (Hodgkin and Huxley 1952).

**Figure 6:**
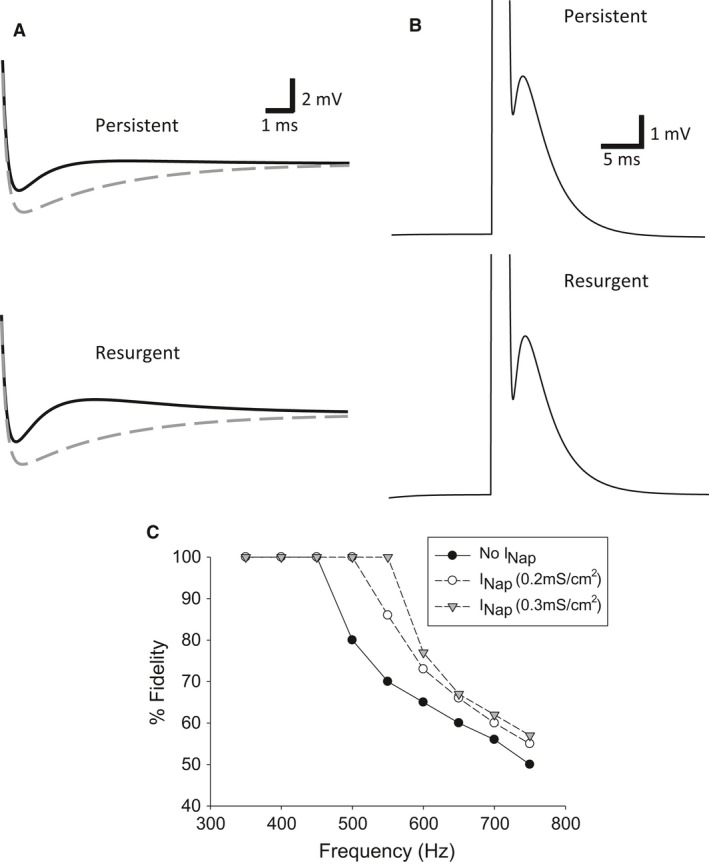
A persistent sodium current decreases AHP duration and increases high‐frequency fidelity in a computational model. (A) Single‐compartment model with either a persistent sodium current (top, black solid) or resurgent sodium current (bottom, black solid) shows the loss of either current can qualitatively mimic the increase in AHP amplitude and recovery time observed after divalent cation exposure. Gray dashed trace represents the base model absent of either the persistent or resurgent sodium current. The persistent and resurgent sodium current had a conductance of 0.2 and 1.0 mS/cm^2^, respectively, and the resting membrane potential was near the leakage current's reversal potential of −65 mV. (B) Hyperpolarizing the model and increasing the persistent and resurgent sodium current to 0.3 and 1.5 mS/cm^2^ in the single‐compartment model could produce an ADP similar in magnitude as previously reported for the DCMD. Hyperpolarization was induced by shifting the reversal potential of the leakage current to −70 mV. (C) Persistent sodium current increases AP fidelity at high frequencies. The axon model was stimulated for 100 APs at the given frequency and fidelity was defined as stimulations eliciting APs. The persistent sodium current had a conductance of 0.2 and 0.3 mS/cm^2^. Similar effects were observed with a resurgent sodium current with conductances of 1.0 and 1.5 mS/cm^2^ (data not shown).

The model had a resting membrane potential of −65 mV, with an AP peak amplitude of 103 mV, and a half‐width of 0.58 msec. The addition of the persistent sodium current with conductance of 0.2 mS/cm^2^ caused a decrease in the AHP amplitude from 3.6 to 1.4 mV and decreased the recovery time from the AHP to baseline membrane potential from 7.1 to 0.55 msec (Fig. 6A). Similarly, the resurgent sodium current with a conductance 1.0 mS/cm^2^ decreased the AHP amplitude to 2.1 mV and the duration to 0.77 msec (Fig. 6A). Qualitatively, both models of the ADP current mimicked the DCMD's change during divalent cation exposure.

We next investigated whether our model was able to produce an ADP with the persistent or resurgent sodium current similar to that observed in the DCMD (Money et al. 2005). We hyperpolarized the resting membrane potential by setting the leakage current's reversal potential to −70 mV and increased the persistent and resurgent sodium conductances to 0.3 and 1.5 mS/cm^2^, respectively. Both had the effect of producing an ADP (Fig. 6B) of similar magnitude to that previously reported in the DCMD (Money et al. 2005). This suggests a single current could be responsible for shortening AHP and producing an ADP.

We then explored the effect shortening the AHP duration had on firing fidelity by challenging the single‐compartment model with a stimulation pattern of 100 spikes at frequencies between 350 and 700 Hz. Fidelity was determined by the number of APs elicited at a given frequency. The persistent sodium current, with a conductance of 0.2 mS/cm^2^, maintained fidelity at 500 Hz and with a conductance of 0.3 mS/cm^2^ could maintain fidelity at 550 Hz. Both conductances improved fidelity at all frequencies >600 Hz compared to the basic model, though they also showed similar reduction near 700 Hz (Fig. 6C). Nearly identical results to the two persistent conductances were obtained with a resurgent sodium conductance at 1.0 and 1.5 mS/cm^2^ (data not shown).

**Figure 7:**
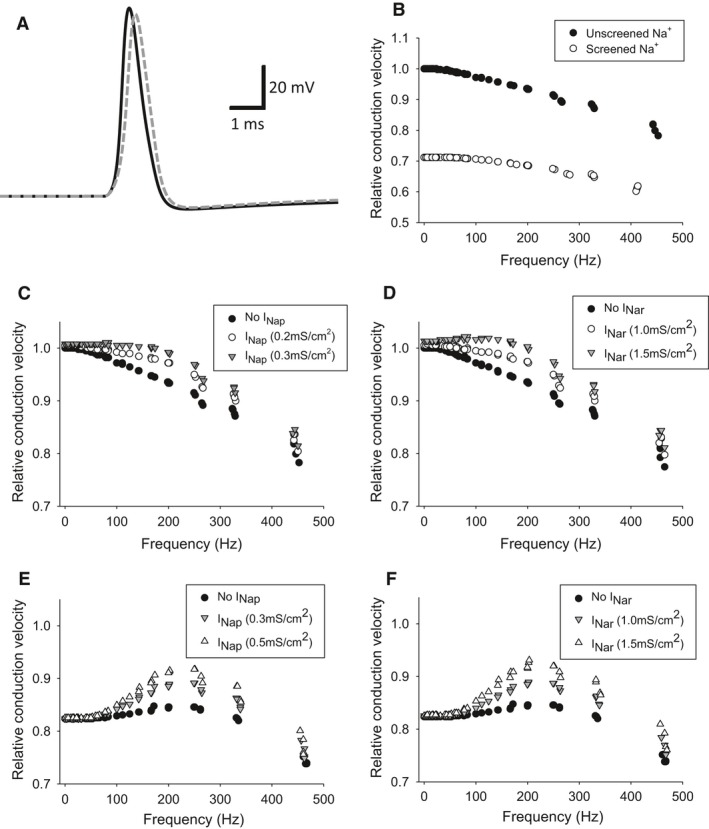
Sodium modulation of CV in a multicompartment model. (A) APs from a multicompartment model of the DCMD axon with (gray dashed) and without (black solid) transient sodium screening. An increase in rise time is clearly observed. (B) The multicompartment model shows a characteristic decline in CV at higher frequencies of up to ~20% (black symbols). Screening of the sodium current caused a decline in CV across all frequencies (open symbols). (C) The addition of the persistent sodium current or (D) resurgent sodium current with increasing maximal conductance increases the CV of higher frequency APs. (ED) Hyperpolarizing the resting membrane potential with a persistent or (F) resurgent sodium current caused an increase in CV of APs around the 200 Hz frequency band. Hyperpolarization was caused by shifting the leakage current's reversal potential to −70 mV. (B–F) Stimulation of the axon model was with a pattern similar to the DCMD's response to a looming visual stimulus. (C–F) Relative conduction velocities were made relative to the first AP's CV in the “No I_N__ap_” condition in C.

We also built a multicompartment model of the DCMD axon to explore mechanisms that can alter CV. The multicompartment model had properties identical to the single‐compartment model except the leakage current's reversal potential was shifted to −60 mV to better reproduce the tail‐off effect of CV at high‐frequencies (Fig. 2A). We challenged our models with stimulation patterns that resembled the DCMD's activity in response to a looming visual stimulus. Initially, we wanted to determine the effect divalent cation screening of the sodium conductances would have on CV in our model axon. To mimic screening, we reduced the voltages used to calculate the transient sodium current by 10 mV which is close to empirical measurements for divalent cation screening (Hille et al. 1975, Frankhauser). As observed in a single‐compartment model (Fig. 7A), the screening resulted in changes to the rising phase of the AP that are similar to those observed during divalent cation exposure (Fig. 3A). For the multicompartment model, we injected 520 nA/cm^2^ into each compartment to compensate for the depolarization caused by the voltage screening. After compensation, there is a 20–25% decrease in relative CV across all frequencies, with low frequencies experiencing a larger decline than high frequencies (Fig. 7B). This confirms our hypothesis that divalent cation screening could contribute to the CV slow‐down observed in the DCMD.

We then explored how AHP shortening could affect conduction. We included the same channel kinetics for either channel from the single‐compartment models and again, challenged the models with a stimulation pattern that resembled the DCMD's activity. Persistent conductances of 0.2 and 0.3 mS/cm^2^ and resurgent conductances of 1.0 and 1.5 mS/cm^2^, produced similar increases in CV, particularly at frequencies >150 Hz (Fig. 7C and D). We then hyperpolarized the resting membrane potential of the models with a shift in the leakage current's reversal potential to −70 mV to produce an ADP, to assess its role in conduction, and scaled CV relative to the same AP as the original model in (Fig. 7C). Addition of the persistent or resurgent sodium channel increased CV of APs with frequencies centered around 250 Hz with little effect on the lowest or highest frequency APs (Fig. 7E and F).

To ensure our results were not dependent on the computational model used, we included the persistent sodium current in two, well‐established axon models: the Hodgkin–Huxley (HH) model of the squid giant axon (Hodgkin and Huxley 1952) and the Connor–Stevens (CS) model of the crab motor axon (Connor and Stevens 1971; Connor et al. 1977). The HH model contains three conductances, similar to our model: a transient sodium, a delayed potassium, and a leakage conductance. The CS model contains similar conductances as the HH model and includes an A‐type potassium channel.

Given the large sodium conductance of both models (HH and CS: *G*_Na_ = 120 mS/cm^2^) the models failed to produce any reduction in CV at high frequencies. This is due to both animals’ (squid and crab) need to function at cooler temperatures expected of an ocean environment. As a result, we reduced the model's temperature coefficient (*φ*) of the channel kinetics from 4, as per our model of the DCMD (Wang (1998)) to 1 and 1.87, which, assuming

(3) $$\phi =3^{(\mathrm{temperature}-6.3)/10}$$

corresponds to temperatures of 6.3°C and 12°C, respectively. As such, we scaled the persistent sodium current's time constant with temperature according to *t*_p_ = 4/*φ*.

**Figure 8:**
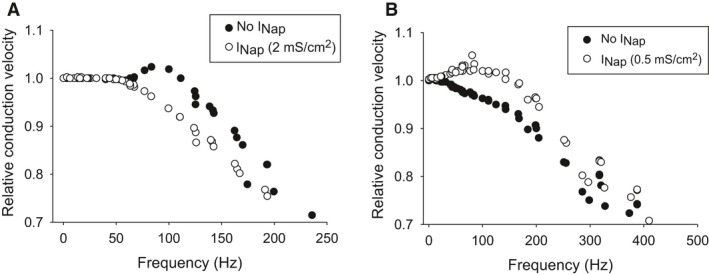
The effects of the persistent sodium current generalize to different models. (A) Simulations from a Hodgkin–Huxley multicompartment model shows a similar increase in CV of high‐frequency action potentials caused by the persistent sodium current. (B) Same as (A), however, with a Connor–Stevens model instead. A similar increase in CV by the persistent sodium current is also observed. (A–B) Both models had a temperature coefficient of 1.87 and were stimulated with a looming stimulus pattern identical to those in Figure 7.

We attempted to adjust the resting membrane potential of the HH model to −60 mV by shifting the leakage conductance, to better compare our model's results with it, however, in doing so produced uncontrolled, repetitive APs. As a result, we maintained the resting membrane potential at −65 mV for all simulations. With *φ* = 1.87, the HH model showed a reduction in CV for high‐frequency APs and was incapable of conducting frequencies >250 Hz (Fig. 8A). The addition of the persistent sodium current with a conductance of 2 mS/cm^2^ increased CV of high‐frequency APs with similar magnitude as our model with the persistent sodium current. Similar results were observed with the persistent sodium conductance when *φ *= 1 (data not shown).

For the CS model, the resting membrane potential is ~−68 mV, partly due to the additional A‐type potassium current. To adjust the resting membrane potential, we increased the leakage conductance's reversal potential from −17 to 9 mV which shifted the resting membrane potential of the model to ~−60 mV. We also simulated the effects of reducing the A‐type potassium current's conductance (from: 47.7 to 29 mS/cm^2^) to shift the resting membrane potential to −60 mV and found qualitatively similar results (data not shown). At *φ *= 1.87, the model was capable of conducting APs >400 Hz and showed a decrease in CV with higher frequencies similar to our DCMD and HH model (Fig. 8B). The addition of the persistent sodium current with maximal conductance of 0.5 mS/cm^2^ increased CV of high‐frequency APs with similar magnitude to both our DCMD and HH model. Similar results were also observed with *φ *= 1 (data not shown).

The observation that two alternative models produce qualitatively similar results indicates our findings are robust and not dependent on the parameters we choose.

## Discussion

We investigated ionic mechanisms in the DCMD axon that allow for faithful high‐frequency transmission and demonstrate that a current exists that shortens the AHP duration. Computational modeling suggests that shortening the AHP duration could increase high‐frequency fidelity and conduction velocity.

### Divalent cations effects on the transient sodium current

The divalent cations effects on the rise time and max rise slope were indicative of modulation of the transient sodium current. This can arise from the surface charge screening effect where divalent cations bind negative surface charges on the plasma membrane or ion channel, distorting the local electric field (Frankenhaeuser and Hodgkin 1957; Hille et al. 1975). This can reduce the voltage difference across the channel, slowing gating properties and decreasing the current conducted by the ion channel. Divalent cations can also directly interact with the activation gate of the transient sodium channel, slowing its activation as well (Gilly and Armstrong 1982b). Surface charge screening and direct slowing of the activation gate would explain the increased rise time we observed in the DCMD. They may also explain why conduction failed over longer exposures as the accumulation of divalent cations further impeded the activation gate eventually causing a conduction block. Modulation of the transient sodium current was likely the primary cause of the divalent cation decreased CV as CV is heavily dependent on active transient sodium channels (Carp et al. 2003; De Col et al. 2008). Our model showed a significant decline in CV following a 10 mV screening of the sodium current that supports this theory, and provides caution for axonal CV studies that involve large changes (~mmol/L) to extracellular divalent cation concentrations. However, our model also suggests a role for persistent or resurgent sodium currents that can fine tune high‐frequency CV.

The differences between cadmium's effects at room and high temperature may be an amplification of the screening effect by the temperature scaling of the channel kinetics. Channel kinetics scale nonlinearly with temperature (for a review see Robertson and Money 2012) and can increase channel rates by a factor of 2.5–4.2 times over the temperature range we used (assuming Q10 of 2–3, respectively). Shifts in the activation curves brought on by the divalent cation screening are then amplified by the temperature scaling, resulting in a larger effect at higher temperatures. This may also explain why the room temperature experiments required longer exposure, to accumulate sufficient divalent cations for the screening effect to be observed.

### Increased CV in calcium‐free saline

There are several explanations for how removal and addition of extracellular calcium could increase and decrease CV, respectively. As a divalent cation, calcium may be screening transient sodium channels, and their removal may increase CV. Although we attempted to control for this effect using magnesium as a substitute ion, magnesium tends to be less potent than other divalent cations, including calcium, at binding negative surface charges and screening (Hille et al. 1975; Hanck and Sheets 1992). Given how the magnitude of the screening effect can vary across the different divalent cations it would be difficult to accurately balance screening effects.

Calcium's effect may have also been through a calcium‐dependent potassium channel that hyperpolarizes the resting membrane potential thereby reducing CV. During heat stress, the resting membrane potential of the DCMD hyperpolarizes (Money et al. 2005) that may be caused by a calcium‐dependent potassium channel and could explain why calcium's effect are absent at room temperature. Increase in intracellular calcium concentration is also known to occur with higher temperatures and plays an important role in conferring tolerance to heat stress at synapses in *Drosophila* (Barclay and Robertson 2003; Klose et al. 2008).

Although calcium saline manipulation affected the DCMD's performance, it did not mimic the effects of the divalent cations on CV. We could be reasonably certain that calcium‐free saline had removed calcium from the DCMD's extracellular space, as high‐temperature effects occurred within 20 min of exposure and were stable up to 50 min past exposure. This is consistent with T‐type calcium channels and calcium‐activated nonselective cation channels (Haj‐Dahmane and Andrade 1997) not being responsible. Furthermore, it remains unlikely that cadmium and nickel were inhibiting a transient receptor potential (TRP) current, which can also shorten AHP duration, as most channel isoforms that carry this current can conduct divalent cations (Bouron et al. 2015) and those isoforms that cannot are activated by cytoplasmic calcium increases (Launay et al. 2002; Hofmann et al. 2003; Lei et al. 2014). Future experiments should focus on rigorously ruling out calcium currents involved in AHP shortening via highly selectively calcium channel blockers.

It's also unlikely the AHP shortening was due to reduction in potassium current brought on by the divalent cations. Divalent cations typically modulate the activation time constant of the delayed‐rectifying potassium channel without a large effect on deactivation (Gilly and Armstrong 1982a; Armstrong and Matteson 1986) which does not explain the AHP increase. Likewise, with A‐type potassium currents, divalent cations shift the inactivation curves in a depolarized manner (Erdelyi 1994) resulting in shortening the AHP, not increasing it. Therefore, we believe the most likely cause of the divalent cations were through a persistent or resurgent sodium current as have been described in several axons (Stys et al. 1993; Crill 1996; Astman et al. 2006; Kim et al. 2010).

### A role for the persistent/resurgent sodium currents

Persistent sodium currents arise from transient sodium channels that open during an AP and remain open past the hyperpolarizing phase of the AP as they fail to inactivate (Crill 1996). By remaining active at hyperpolarized potentials, they provide a depolarizing potential for high‐frequency and repetitive firing (Harvey et al. 2006). Resurgent sodium currents occur when transient sodium channels reactivate late in the AP and remain active into the hyperpolarizing phase until inactivated and are also involved with high frequency and repetitive firing (Khaliq et al. 2003).

In axons, persistent sodium currents increase excitability (McIntyre et al. 2002), and resurgent sodium currents secure high‐frequency fidelity in calyx of Held (Kim et al. 2010). Our model supports both functions for the persistent and resurgent sodium current and suggests a possible mechanism to decrease expensive, high‐frequency signaling that occurs in the DCMD after metabolic stress. After anoxic stress, the DCMD axon conducts fewer and slower high‐frequency APs (Money et al. 2014). However, under hypoxic conditions, persistent sodium currents increase in hippocampal (Hammarstrom and Gage 2000) and cardiac tissue (Ju et al. 1996) that should increase excitability, which conflicts with the DCMD's decreased excitability during hypoxia (Money et al. 2014). It is possible the persistent sodium currents are more tightly regulated in the DCMD given that it can fully recover from anoxia, whereas hippocampal and cardiac tissue experience cell injury and death. Also, elevated cAMP levels can increase persistent sodium currents (Nikitin et al. 2006) and may account for the DCMD's recovery of CV from hypoxia after application of adenylate cyclase activator (Money et al. 2014).

A characteristic of neurons with persistent and resurgent sodium currents is an ability to cause bursting activity. In neocortex (van Drongelen et al. 2006), the pre‐Bӧtzinger complex (Thoby‐Brisson and Ramirez 2001; Del Negro et al. 2002), cerebral snail (Nikitin et al. 2006) and leech heart interneurons (Opdyke and Calabrese 1994), persistent sodium currents underlie intrinsic and network bursting, while resurgent sodium currents are involved in bursting activity of cerebellar Purkinje cells (Raman and Bean 1997; Khaliq et al. 2003). In the DCMD, rigorous quantification of its activity in response to a looming visual stimulus reveals robust high‐frequency bursting that increases as the visual stimulus approaches (McMillan and Gray 2015). It is possible that a persistent or resurgent sodium current allows faithful transmission of high‐frequency bursts by the DCMD from the LGMD to its postsynaptic partners. Burst generation is commonly observed in sensory pathways as a means of enhancing the signal‐to‐noise ratio at synapses, causing a greater release of neurotransmitter (Krahe and Gabbiani 2004), which is exemplified in the DCMD axon, as several high‐frequency APs are required to elicit activity in a post‐synaptic interneuron (Santer et al. 2006). For bursting neurons that require high‐fidelity transmission of high‐frequency bursts, including the thalamic relay interneurons (Steriade et al. 1993) and cricket auditory neurons (Marsat and Pollack 2006), our data suggest that AHP shortening can increase CV for high‐frequency APs which may assist in maintaining fidelity during high‐frequency bursting. Also, we are the first to demonstrate, to the best of our knowledge, that a persistent or resurgent sodium current can reduce the subnormal conduction of high frequency APs in a computational model, which would maintain the temporal fidelity within a burst of APs during transmission. These computational results may shed light on the functional role of persistent or resurgent sodium currents in other axonal models including optic nerve axons (Stys et al. 1993) and at the calyx of Held (Kim et al. 2010).

### ADP and heat stress

Although we did not show that the current responsible for AHP reduction is involved in the ADP formation at high temperature in the DCMD, our computational modeling suggests this is possible. So what is the functional role of an ADP‐causing current at high temperature in the DCMD? In the DCMD axon, elevated temperatures result in a reduction in excitability that could be explained by the accompanying hyperpolarization of the membrane, as the magnitude to reach the threshold for AP initiation has increased (Money et al. 2005). In heat‐shocked animals hyperpolarization at high temperatures is larger than control animals, however, they exhibit an increase in membrane excitability (Money et al. 2005). Hyperpolarization may be a protective mechanism from thermal stress as heat‐shocked locusts experience heat‐induced conduction failure in the DCMD at higher temperatures (Money et al. 2009). Heat‐induced conduction failure occurs with an abrupt increase in extracellular potassium (Money et al. 2009) and may be due to temperature mismatch between neural activity and potassium clearance mechanisms (Robertson and Money 2012). Hou et al. (2014) found locust thoracic ganglia increase the number of Na^+^/K^+^ ATPase in neuronal plasma membranes after heat‐shock, presumably to improve potassium clearance. This could account for the increased hyperpolarization in the DCMD after heat‐shock if the axon increased the number of Na^+^/K^+^ ATPase in its plasma membrane. The ADP, which is enhanced in heat‐shocked animals (Money et al. 2005), then provides a temporary depolarization to increase excitability of high‐frequency APs, as predicted by our model (Fig. 5E), which trigger escape responses (Santer et al. 2006; Fotowat et al. 2011) without compromising the protective effect of hyperpolarization. Future experiments should determine if the ADP modulates high‐frequency CV of the DCMD axon and if so, in what frequency band.

### Model limitations

Like all computational models, ours have several limitations, including the absence of electrophysiological data characterizing the sodium, potassium, or leakage currents in the DCMD. Channel kinetic data for large axons are distorted by incomplete space clamps while fitting channel kinetics by AP waveform can produce endless combinations (Sengupta et al. 2010). Also, the model assumes a fixed radial geometry for the DCMD, which is incorrect, as tapering and branch points occur throughout the axon (O'Shea et al. 1974). However, we feel the effects of both limitations are minimal as we were more interested in the AHP's role in conduction rather than an accurate description of the DCMD axon. Also, our results for the persistent sodium current extended to two different models with similar results. Another limitation in the model is the ADP produced. Though similar in magnitude to the ADP described by Money et al. (2005), it decays quicker, which could be attributed to the short time constant for the persistent and resurgent sodium current. By increasing the time constant, we could increase the duration the persistent or resurgent sodium current is active and therefore increase the ADP's time course. However, this was not undertaken in our study.

Our model also assumes the presence of a single potassium current even though many potassium channel subtypes have been identified in axons (Debanne 2004). However, we feel a single potassium current is sufficient to capture high‐frequency firing in the DCMD neuron. We know activity‐dependent hyperpolarization of the resting membrane potential of the axon is primarily contributed by the Na^+^/K^+^ ATPase (Money et al. 2014), which suggests that BK and SK channels are absent. Also, to test for a potential interaction of slow‐activating potassium currents with the persistent or resurgent sodium current, the Connor–Steven's model with an A‐type potassium current was included and this still shows similar effects of the persistent and resurgent sodium current on CV profile. Furthermore, given the fast firing frequencies we were interested in (>100 Hz primarily) potassium channels with short time constants would have the largest effects in shaping the CV. K_v_3 potassium channels have short time constants and contribute to high firing frequency in other neurons (Rudy et al. 1999; Rudy and McBain 2001), however, they are TEA‐sensitive and our unpublished observations with TEA in the DCMD found no effect. Although this does not rule out all possible potassium channels and dynamics, our use of a single potassium current sufficiently captures the frequency‐dependent effects on CV. Furthermore, our results with the persistent or resurgent sodium current matches well with other models that include more complex potassium dynamics (D'Angelo et al. 2001; McIntyre et al. 2002; Kim et al. 2010). Future models of the DCMD may partition the single potassium current into several to observe their interactions with the persistent and resurgent sodium current which could be insightful for interpreting heat shock in locusts as potassium currents are known to be downregulated by this process (Ramirez et al. 1999).

Our models also focused exclusively on the persistent or resurgent sodium current even though many different mechanisms could shorten the AHP. However, currently the multicompartment models are computationally intensive, so we focused on the candidate mechanisms we felt were the most plausible: the persistent and resurgent sodium currents. Future simulations should focus on whether similar effects could be achieved with functionally similar channels including a T‐type calcium current.

## Conclusion

Axons demonstrate frequency‐dependent effects on CV that can modulate spike timing. For high‐frequency firing neurons, such as fast‐spiking interneurons and thalamic relay neurons, this effect is potentially more disruptive as subnormal CV increases in magnitude with higher frequencies. Our data show a potential role for mechanisms that shorten the AHP duration, counteract subnormal CV at higher frequencies and improve transmission fidelity.

## Conflict of Interest

None declared.
